# Influence of Selective Carbon 1s Excitation on Auger–Meitner Decay in the ESCA Molecule

**DOI:** 10.1021/acs.jpclett.3c03611

**Published:** 2024-04-12

**Authors:** A. E. A. Fouda, V. Lindblom, S. H. Southworth, G. Doumy, P. J. Ho, L. Young, L. Cheng, S. L. Sorensen

**Affiliations:** † Chemical Sciences and Engineering Division, Argonne National Laboratory, 9700 S. Cass Avenue, Lemont, Illinois 60439, United States; ‡ Department of Physics and James Franck Institute, The University of Chicago, Chicago, Illinois 60637, United States; § Department of Physics, Lund University, Box 118, 22100 Lund, Sweden; ∥ Department of Chemistry, Johns Hopkins University, 3400 North Charles St, Baltimore, Maryland 21218, United States

## Abstract

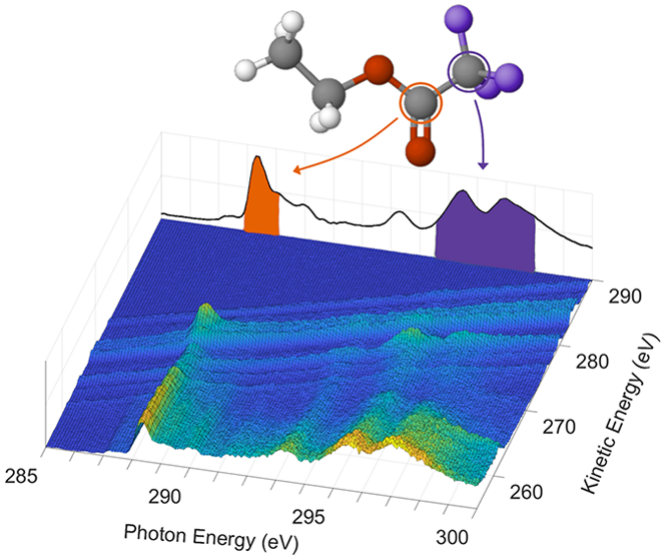

Two-dimensional spectral mapping is used to visualize how resonant Auger–Meitner spectra are influenced by the site of the initial core–electron excitation and the symmetry of the core-excited state in the trifluoroethyl acetate molecule (ESCA). We observe a significant enhancement of electron yield for excitation of the COO 1s → π* and CF_3_ 1s → σ* resonances unlike excitation at resonances involving the CH_3_ and CH_2_ sites. The CF_3_ 1s → π* and CF_3_ 1s → σ* resonance spectra are very different from each other, with the latter populating most valence states equally. Two complementary electronic structure calculations for the photoelectron cross section and Auger–Meitner intensity are shown to effectively reproduce the site- and state-selective nature of the resonant enhancement features. The site of the core–electron excitation and the respective final state hole locality increase the sensistivity of the photoelectron signal at specific functional group sites. This showcases resonant Auger–Meitner decay as a potentially powerful tool for selectively probing structural changes at specific functional group sites of polyatomic molecules.

The phenomenon commonly referred to as the “chemical shift” originates from the binding energy of inner-shell electrons’ sensitivity to the local chemical environment. It implies that X-ray photoelectron spectroscopy (XPS) is a unique tool for the quantitative exploration of conformational changes,^1^ surface chemistry,^2^ and structure in nanoscale materials.^3^ This effect has long been showcased by ethyl trifluoroacetate,^1^ C_4_H_5_O_2_F_3_, commonly referred to as the “electron spectroscopy for chemical analysis (ESCA) molecule”, with four carbon atoms each located in distinct chemical environments. This unique configuration makes it an ideal system to exploit the chemical shift to explore how localized electronic states influence the evolution of the system. However, in the case of inner-shell photoionization, it was shown that Auger–Meitner (AM) decay populates identical dication states in the ESCA molecule irrespective of the site of the core–hole^4^ and that fragmentation patterns are not affected significantly by which carbon site is core-ionized, making a clear case that all memory of the original core–hole site is lost in the AM transition.^5^

The C 1s near-edge X-ray absorption fine structure (NEXAFS) spectrum of the ESCA molecule was recently analyzed, and spectral features associated with resonances at each of the four carbon sites were identified, including three intense resonant features originating at the CF_3_ site.^6^ Several studies have explored the site selectivity in the resonant AM decay following core excitation, based on factors such as the chemical environment, the energy of the excited state, or its symmetry, benchmarked by the experiments outlined in refs (7−9). A recent study on HNCO by Holzmeier et al. found only minor differences in resonant AM spectra for excitation at C, N, or O sites, or for different intermediate states,^10^ while de Moura et al. found that excitation at different nitrogen sites in phthalocyanine leads to visible changes in the spectra.^11^ These examples highlight the complexity of resonant photoemission and that there are often subtle factors which determine the probability for populating final valence states even when site selectivity is possible. By recording the AM spectrum as a function of incident photon energy over the range of the NEXAFS spectrum, once again this work shows that the ESCA molecule can showcase site-dependent transitions, and for a single carbon site we can study how the symmetry of the excited valence state affects both participator [also called one-hole (1*h*)] and spectator [two-hole one-particle (2*h*-1*p*)] decay processes.

In order to address these questions, our study adopts a combined theoretical and experimental approach. We interpret the experimental spectra by calculating transition rates for 1*h* and 2*h*-1*p* electronic states for both direct valence and resonant ionization. We find significant resonant enhancement at particular states when exciting the COO and CF_3_ carbon inner-shell electrons, with a somewhat lesser effect observed for the CH_2_ and CH_3_ sites. For excitation of the CF_3_ electron we find both strong participator decay for the excitation to the π* state and predominantly spectator decay after resonant excitation to valence states of σ* character. Our findings show that significant enhancement of features occurs when both the core-excited and final-state hole orbitals are spatially local to the absorbing atom site. This demonstrates a high sensitivity of the resonant AM decay signal to specific structural regions of polyatomic molecules. This could enable functional group selectivity in future time-resolved resonant AM decay studies of excited-state dynamics.^12−14^

The experiment was performed on the FlexPES beamline at the MAX IV synchrotron radiation facility in Lund, Sweden.^15^ Electron spectra were measured using a VG R4000 Scienta electron analyzer with the spectrometer lens mounted at 54.7° with respect to the plane of polarization.^16^ Valence photoelectron spectra were measured with a total resolution of 45 meV. The electron spectra were recorded with 130 meV resolution. The kinetic energy was calibrated by recording the Ar L-MM AM and 2p photoelectron spectra at a 290 eV photon energy. The Ar L-MM energies were taken from Pulkkinen et al.^17^ The photon energy was calibrated as described in ref (6). The electron maps were corrected for the photon flux measured by using an AXUV 100 photodiode from IRD. No second-order features were visible. Valence electron spectra in Figure 2 are corrected for relative photon flux.

Two complementary levels of theory are used to capture the separate valence photoelectron and AM electron contributions to the spectrum. Here, we briefly summarize these calculations; more detailed descriptions are found in the Supporting Information.

The valence photoelectron calculations extend previous work using equation-of-motion coupled-cluster singles and doubles (EOM-CCSD)^18^ to model the NEXAFS of the ESCA molecule.^6^ EOM-CCSD Dyson orbitals are computed in the CFOUR program^19^ and used with the ezDyson software^20,21^ to compute the cross section using radial Coulomb waves.^22,23^ This approach goes beyond approximating the cross section with Dyson orbital norms,^24,25^ which neglects the interaction of the outgoing electron with molecular frame [sudden approximation (SA)] and is known to describe the cross sections of low kinetic energy poorly. However, the single point charge approximation is less rigorous than accurate multicentered B-spline techniques providing a detailed description of the molecular frame effects by defining a new multicentered basis for the outgoing electron wave function.^26^

AM decay is challenging for accurate theoretical methods; it is a two-electron process involving the ejection of an electron into the continuum. Recently, methods using the OpenMolcas^27^ implementation of the multireference restricted active space self-consistent field (RASSCF) method^28,29^ with second-order perturbation theory (RASPT2) to include dynamic correlation corrections to the energies^30^ have been developed. This includes the spherical continuum for ionization (SCI) approach developed by Grell et al. which describes the continuum using a spherically averaged potential of the bound state cation.^31,32^ Tenorio et al. also implemented the one-center approximation using atomic integrals for the continuum with RASSCF/PT2 calculations, yielding comparable results to the SCI method at a reduced cost.^33^ Methods neglecting the continuum by an electron population analysis add no additional cost to the electronic structure calculation and are attractive for larger systems and for probing ultrafast dynamics with time-resolved AM decay.^12^ Recently, de Moura et al. successfully interpreted the large trans-H_2_-phthalocyanine molecule decay using a population analysis approach.^11^ Here we use the method developed by Mittani et al.,^34^ where the AM intensity is approximated by the electronic populations of the molecular orbitals on the core–hole atom in the core-excited state wave function and is described in detail elsewhere.^34−36^ This approach is similar to the one employed by de Moura et al.; however, it in neglects the lifetime interference contributions which were found to have a minor influence on the results.^11^

In order to correctly account for the competing core-excitation and direct valence ionization processes produced by complementary levels of theory, the separate theoretical contributions were normalized to 1. Then the resonant AM spectra were scaled by a factor derived from the ratio of the total valence ezDyson photoelectron cross section and the EOM-CCSD core-excitation cross section from a previous calculation.^6^ The scaling factor is constant for final states for a given core excitation; the formula and values used are given in the Supporting Information.

In Figure 1b, we show the 2D electron emission map measured as we scan the photon energy through the resonance region near the C 1s thresholds for the four carbon atoms in the ESCA molecule. In the map the linear dispersion of the one-hole final states is clearly visible as the direct valence ionization appears as diagonal lines starting at 285 eV photon energy. The intensity of the direct photoionization is essentially constant over this photon energy range. The map also clearly highlights the resonant contributions to final states at the photon energies corresponding to resonances. In Figure 1a the integrated electron intensity at each photon energy is plotted, indicating the intensity of resonances for excitation of C 1s electrons from the different color-coded sites in the ESCA molecule.^6^ Valence states are populated via direct photoionization and decay of the core-excited states. For example, the outermost valence state appearing at 274 eV kinetic energy exhibits only weak enhancement at the resonances visible in Figure 1a while the states at slightly lower kinetic energy are significantly enhanced at the most intense resonance energies.

**Figure 1:**
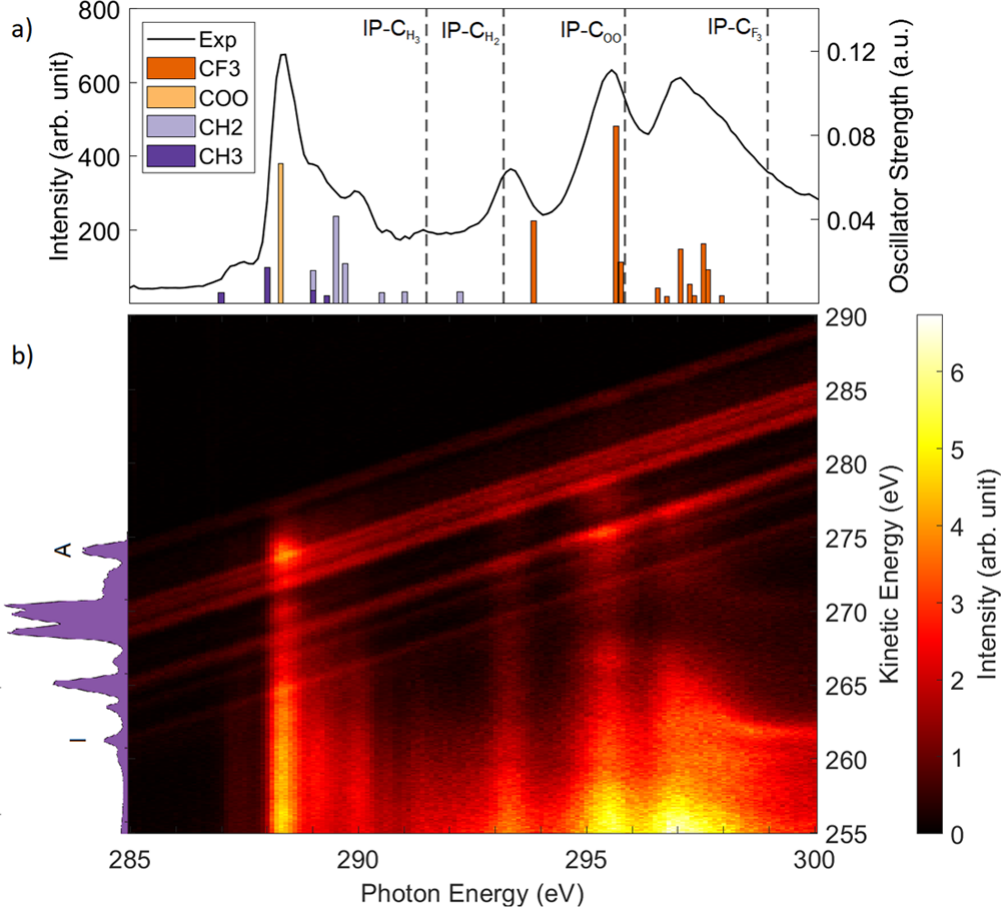
Electron yield spectrum (a) and 2D AM map (b) obtained by measuring electron spectra at photon energy intervals of 100 meV across the resonance range. The spectrum in the top panel contains the integrated electron intensity at each photon energy (left axis). Calculated oscillator strengths (right axis) for transitions from the four carbon sites are shown with bars in (a).^6^ In the 2D map, the electron spectra are presented as the photon energy is scanned through the resonance region. The electron spectrum placed on the left-hand edge of the 2D plot highlights the direct valence contributions (seen as diagonal features in the plot). The color bar on the right indicates the electron intensity.

We will analyze the direct valence photoelectron and resonant participator AM contributions to the spectra separately in Figure 1b, while the resonant spectator contributions are presented in the SM. The valence electronic states are identified in the 120 eV photoelectron spectrum; then, theoretical calculations analyze the resonance features to unravel how the changes in spectral line shapes relate to the original core–electron excitation site and the molecular hole orbitals in the final states.

Figure 2 shows a comparison between theory and experiment for the valence photoelectron spectra measured at photon energies of 120 and 265 eV; no previous measurements of this spectrum have been reported in the literature. The binding energies of the ionized molecular orbitals have been calculated at the EOM-CCSD/cc-pVTZ level using the CFOUR code^19^ (see Table 1), and the cross sections are obtained with the ezDyson code.^21,23^ We find excellent agreement between the experimental and the calculated spectra. This enables us to identify the participating molecular orbitals, which are labeled A–J in Figure 2a. These labels are also used to classify the individual transitions in Table 1. If multiple states lie under the same peak, they are labeled A1, A2, etc. The Dyson orbitals for each transition are given in the left column of the orbital tables in the Supporting Information. We note that for the two highest binding energy peaks I and J, there is a small overestimation in the EOM-CCSD binding energies, which is expected for high-energy valence cation states in larger systems where the molecular orbital picture is less valid.^37^ However, our results show that EOM-CCSD sufficiently describes the electron-correlation effects in photoionization processes for all the cation states in the molecule. This is not guaranteed by density functional theory based methods that optimize the cation wave functions with an overlap criterion.^11^

**Figure 2:**
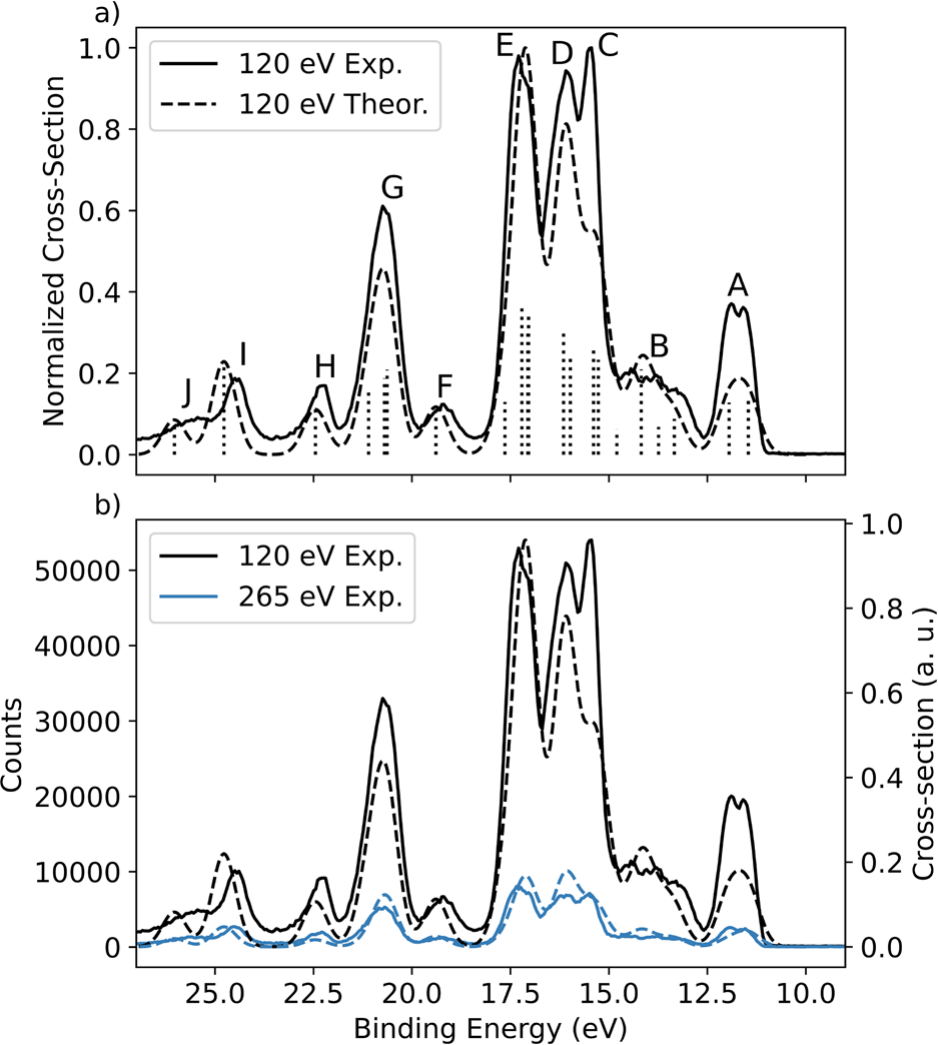
Comparison between experiment and theory for the direct valence photoionization spectrum of the ESCA molecule. (a) compares the CSSD calculated spectra with ezDyson cross sections^21,23^ to the experiment at 120 eV. Peaks have been labeled A–J, and the calculated ionization energies, Dyson orbital norms, and cross sections are given in Table 1. (b) Comparison of the change in cross section between 120 and 265 eV between the experiment and ezDyson spectrum. The experimental spectra are scaled according to the photon flux.

**Table 1:** Direct Valence Photoionization Energies (eV), Dyson Orbital Norms (Sudden Approximation Cross Section), and the 120 and 265 eV ezDyson Cross Sections (a.u.) of the ESCA Molecule Calculated by EOM-CCSD

state^a^	binding energy (eV)	Dyson norm	σ 120 eV (a.u)	σ 265 eV (a.u)
A1	11.46	0.943	0.129	0.033
A2	11.95	0.958	0.123	0.017
B1	13.34	0.943	0.085	0.019
B2	13.74	0.965	0.066	0.009
B3	14.18	0.945	0.201	0.037
B4	14.80	0.950	0.060	0.013
C1	15.27	0.957	0.224	0.023
C2	15.39	0.954	0.258	0.077
D1	15.98	0.944	0.230	0.069
D2	16.15	0.939	0.260	0.056
D3	16.15	0.958	0.291	0.055
E1	17.04	0.935	0.328	0.059
E2	17.06	0.958	0.286	0.053
E3	17.21	0.931	0.348	0.057
E4	17.64	0.953	0.125	0.019
F1	19.39	0.936	0.112	0.025
G1	20.63	0.945	0.201	0.063
G2	20.70	0.955	0.180	0.055
G3	21.10	0.938	0.150	0.017
H1	22.45	0.995	0.107	0.017
I1	24.77	0.919	0.220	0.047
J1	26.03	0.875	0.082	0.019

a States have been assigned with respect to the peak assignments given in Figure 2.

Figure 2a shows that the theory correctly identifies peaks E, D, and C as the major contributions and correctly describes the relative shape across the spectrum. This result suggests that the ezDyson method is a promising approach for the challenging simulation of mid-sized (>10 atoms) polyatomic molecular photoelectron spectra, with few examples in the literature.^37−40^ The ezDyson calculation considers the potential of the molecular frame only as a point charge of +1 centered at the centroid of the contributing Dyson orbital. The strength of this approximation is further demonstrated by Figure 2b, which compares valence photoelectron spectra between 120 and 265 eV. The calculation captures the reduction in the experimental cross section with excellent agreement. This indicates that electrons with kinetic energies around 100 eV have little interaction with the molecular frame of the ESCA molecule. This is further supported by the good experimental agreement of the SA spectra shown in the SM (Dyson orbital norms are given in Table 1). Approximations for the cross section with EOM-CCSD Dyson orbitals or Coulomb waves vastly reduce the computational effort and complexity of more sophisticated methods.^26^ This suggests that they are suitable approaches for simulating time-resolved photoelectron spectroscopy experiments that probe ultrafast dynamics,^41^ which requires spectral computation across numerous conformers.^25^

The 2D electron emission map in Figure 1 illustrates differences in the intensities of the 1*h* final states. To investigate these differences further, we focused our analysis on specific electron spectra corresponding to intense and relatively isolated resonance features, indicated by color-coded bars in Figure 1a. Specifically, we examined the COO 1s → π* resonance at 288.3 eV, the CF_3_ 1s → π* resonance at 293.3 eV, and the CF_3_ 1s → σ* resonance at 295.6 eV, as reported by Sorensen et al.^6^ These spectra are shown in Figure 3 on a binding energy scale together with the nonresonant spectrum. A comparison with the off-resonance spectrum reveals that all features observed in the direct valence spectrum are enhanced during resonance. However, different excitation energies exhibit noticeably distinct enhancements. The most striking features occur in the 11–16 eV binding energy range for the COO 1s → π* excitation (288.3 eV) and around 21 eV for the CF_3_ 1s → σ* resonance (295.6 eV). We denote these regions as resonant features X and Y, respectively. Interestingly, for the COO excitation at 288.3 eV, the 2*h*-1*p* states above 21 eV are also significantly enhanced. However, the spectral line shape for the CF_3_ 1s → π* resonance exhibits minimal changes compared to the off-resonant signal, as illustrated in Figure 3.

**Figure 3:**
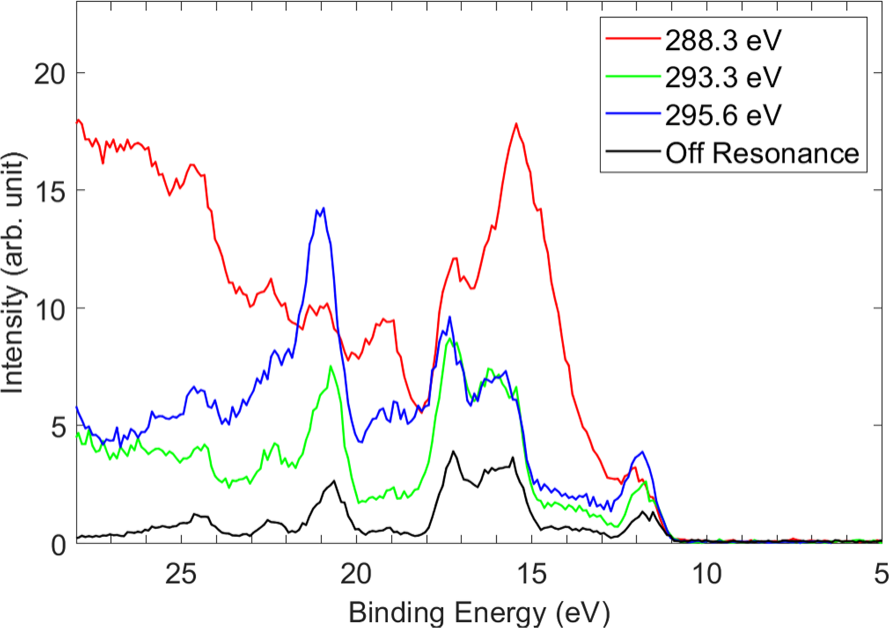
Resonant AM spectra measured at 288.3, 293.3, and 295.6 eV are presented on a binding energy scale together with the direct valence spectrum measured at 265 eV. Each spectrum was summed over a width of ±0.2 eV.

In Figure 4a, the experimental 2D spectrum is compared to the calculated 1*h* spectrum, which includes valence photoelectron and participator AM final states. Features X and Y are highlighted in the comparison. In Figure 4b, the experimental spectra measured at the COO 1s → π* and CF_3_ 1s → σ* resonances are presented alongside the calculated direct and resonant final 1*h* state spectra. The green and orange sticks show the respective participator AM and valence photoelectron contributions to the theoretical spectra, calculated by the RASSCF/PT2 and EOM-CCSD levels of theory, respectively (see the Supporting Information for details). We note that although the 1*h* final states appearing in the direct and resonant spectra represent the same set of ground-state orbitals, their descriptions and binding energies differ slightly between the different levels of theory used (inducing a minor artificial broadening to the theoretical line shape in Figure 4). As accurate and scalable calculations simulating both phenomena under a common theoretical description are scarcely available, we show that two different levels of theory for the separate direct ionization and resonant AM contributions provide a complementary analysis of the experiment. In the SM we provide a 1-to-1 mapping between the orbital shapes from the different calculations, where the RASSCF 1*h* natural orbitals from the separate COO 1s → π* and CF_3_ 1s → σ* calculations are shown in the tables containing the EOM-CCSD Dyson orbitals. These natural orbitals result from the diagonalization of the first-order density matrix and represent linear combinations of 1*h* Slater determinants optimized during the RASSCF calculation.

**Figure 4:**
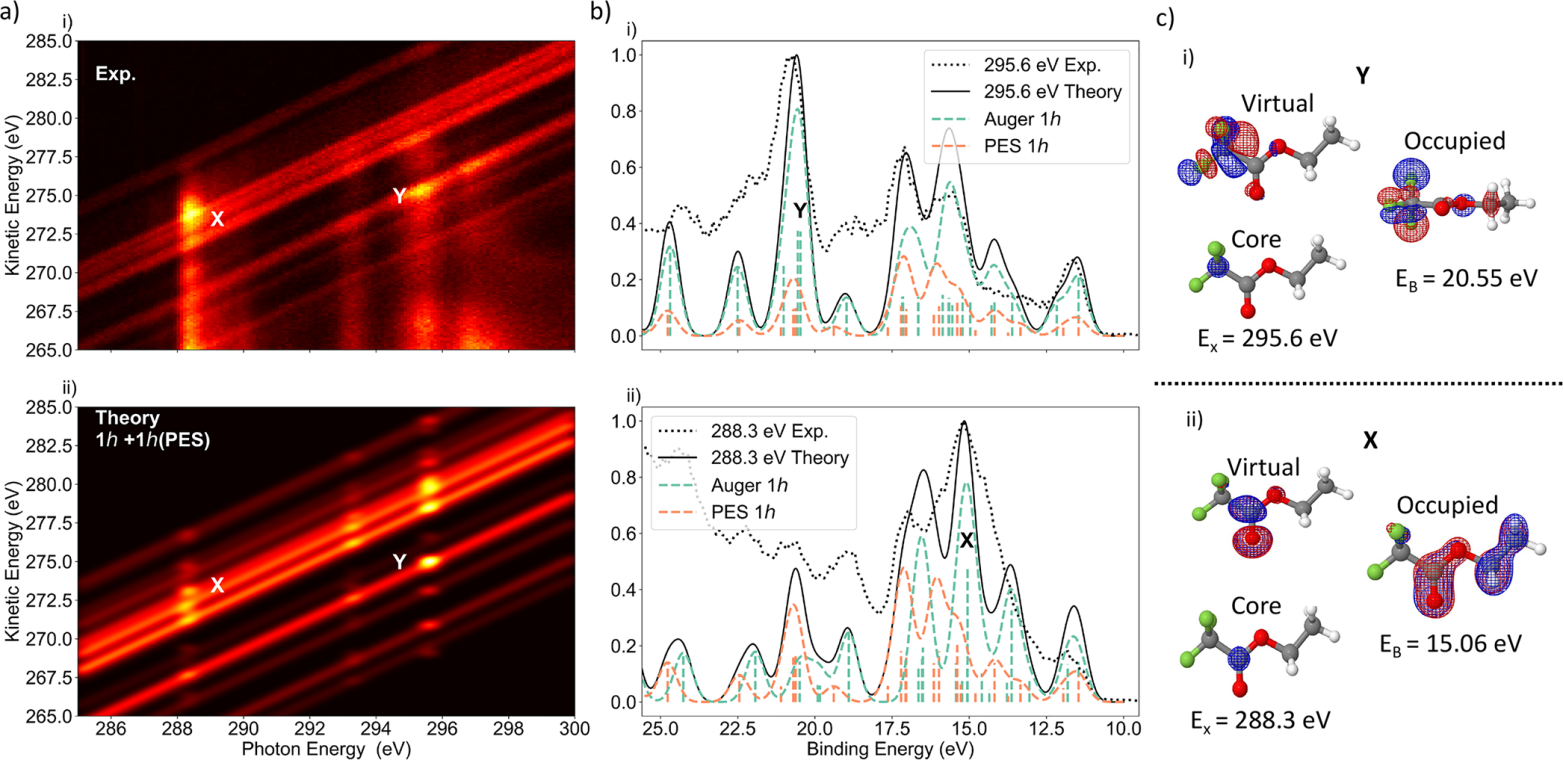
Theoretical analysis of the resonant enhancement features due to participator AM decay channels observed in the experimental spectrum. (a) (i, ii) Experimental and theoretical 2D AM maps. The theory map contains calculated resonant photoelectron spectra at 288.3, 293.3, and 295.6 eV. The theoretical off-resonant lines were calculated at 288.3 eV by EOM-CCSD. (b) (i, ii) Experimental (black dotted) and theoretical (black solid) 1D spectra at photon energies 288.3 and 295.6 eV. The theoretical resonant spectra (black dashed) are a combination of the RASSCF/PT2 calculated AM participator (Auger 1*h*) channels (green dashed) and the EOM-CCSD ezDyson calculated direct photoionization (PES 1*h*) channels (orange dashed). (c) (i, ii) RASSCF natural orbitals involved in the resonant enhancement regions X and Y. The calculated Auger 1*h* spectra were shifted so the lowest energy binding peak was aligned to the calculated PES 1*h* spectra by 0.05 eV at 288.3 eV, 0.06 eV for 293.3 eV, and 0.8 eV for 295.6 eV.

Despite that the AM decay calculations exclude the continuum electron wave function, Figure 4a shows that they effectively capture both features X and Y and the absence of orbital-specific enhancement at the 293.30 eV resonance very well. Here, we aim to explain the presence of features X and Y by discussing two factors related to the site and state selectivity of resonant AM decay: (i) the strength of the core excitation and (ii) the overlap of the ground-state occupied orbitals with respect to the core–hole site.

Substantial resonance intensity is a prerequisite for detecting a significant change in the spectrum and is evident in the experimental map in Figure 1 where strong resonant features translate into higher intensity in the 2D map. However, if this was the sole factor behind resonant enhancement of features X and Y, then similar features would be expected to occur at all significant resonances in Figure 1, such as the CH_2_ and CF_3_ 1s → π* resonances at 290 and 293 eV, which is not the case. Therefore, features X and Y can not only be rationalized by the intensity alone but require a closer study of the orbitals.

State-selective enhancement is most clearly observed for feature Y, and Figure 4b(i) shows that there are three final 1*h* states under this peak as identified in Figure 2 and Table 1. The natural orbital of the state at 20.55 eV is shown in Figure 4c(i). Visual inspection of this and the other two natural hole orbitals (Supporting Information) reveals a significant spatial overlap with the CF_3_ core–hole site. This is experimentally verified in Figure 5, which shows the experimental intensities of the valence states B, D, F, and G (Figure 2 and Table 1) plotted as a function of photon energy. Figure 5 exhibits a peak in the yield for the states associated with peak G at a 295.6 eV photon energy.

**Figure 5:**
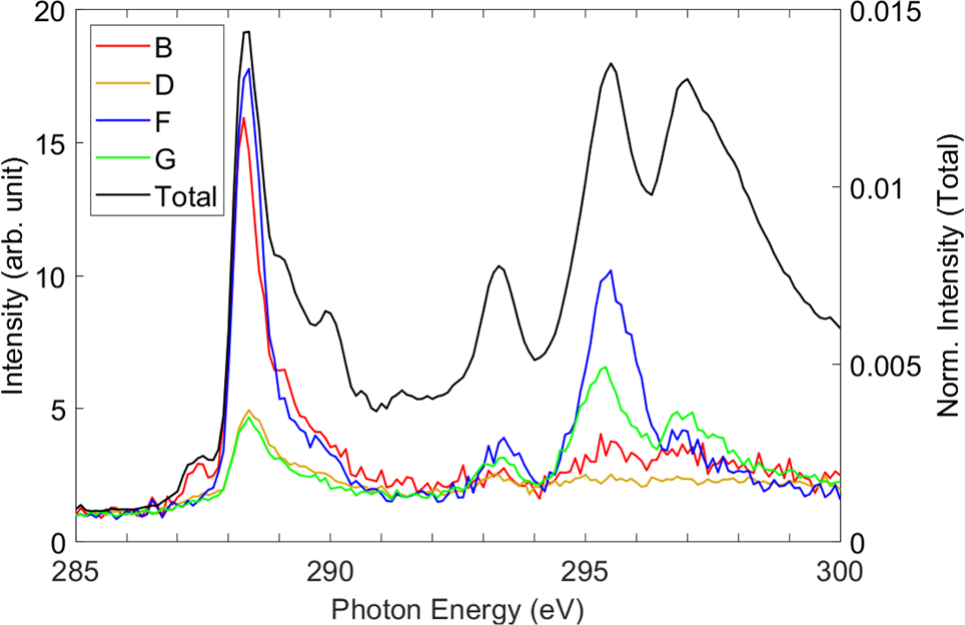
Intensity of valence states B, D, F, and G plotted as a function of photon energy. The labels in the legend correspond to those in Table 1. The partial yields are scaled for equal intensity at 285 eV. The total yield from integration of all measured electrons is shown in the plot with the same scaling.

Feature X at the 288.3 eV resonance is seen over a broader *E*_B_ range (13–18 eV) than Y and is therefore more complex to interpret as it involves a larger number of contributing final 1*h* states. This resonance is dominated by the COO 1s → π* excitation; the Supporting Information orbital tables show that, generally, the natural orbitals in this *E*_B_ range are not all spatially confined to the COO site. However, Figure 4 c(ii) shows that the natural hole orbital at the enhancement maximum binding energy (15.06 eV) does strongly overlap with the COO core–hole site. However, though feature X is less spatially selective than Y, its increased strength can be attributed to the involvement of a larger number of contributing final states. This is reflected by the experiment in Figure 5 which principally shows peaks for 1*h* final states B and F, with smaller contributions from states D and G at 288.3 eV.

Figure 5 reiterates the importance of the core-excitation strength in determining the degree of AM decay. The intensities of the four 1*h* states are shown as a function of photon energy. Excitation of a C 1s electron at the CH_2_/CH_3_ site (287 eV) results in the enhancement of state B. At the 288.3 eV peak (COO 1s → π*) the same state is strongly enhanced in addition to state F, while states D and G increase only slightly. For CF_3_-based excited states that we observe at the CF_3_ 1s → π* state at 293.3 eV, all four states exhibit enhancement. At 295 eV (the CF_3_ 1s → σ* state), states F and G experience a significant increase in intensity, while state B shows a moderate increase. At 297 eV (CF_3_ 1s → 3p), the state labeled G in Table 1 is strongly enhanced; both states B and F also show increased intensity. These trends highlight that apart from the excitation site, the molecular orbitals of the final states significantly influence the results.

A significant contribution of the electron yield originates from spectator (2*h*-1*p*) final states. Figure 1b shows that at each resonance the enhancements are observed at kinetic energies below 265 eV, which mostly correspond to the 2*h*-1*p* final states. While this signal still depends on the core-excitation strength it will be more reliant on the overlap of the occupied valence orbitals to core–hole site. This is clearly observed between the COO 1s → π* and CF_3_ 1s → σ* spectra shown in Figures 1 and 5; the former overlaps with more occupied valence orbitals (discussed in Figure 4) and has a stronger and broader signal in this region. At kinetic energies below 265 eV, the observed increase in the photoelectron signal with increasing photon energy is possibly due to additional contributions from nonresonant AM decay, which would increase as the photon energy passes more C 1s ionization potentials (Figure 1a). The kinetic energies for the nonresonant AM decay electrons begin at 260 eV and therefore overlap with the 2*h*-1*p* decay channel.^4^ In the SM we include the 2*h*-1*p* final states in the theoretical resonant spectra shown in Figure 4. At a photon energy of 288.3 eV, the inclusion of the 2*h*-1*p* final states improves the overall agreement with experimental data, particularly for binding energies above 18 eV. At the 295.6 eV resonance the calculation shows there is little spectator contribution to the intensity in the shown binding energy region of the spectrum, and the experimental line shape is well represented by the final 1*h* states alone.

In conclusion, we have analyzed the evolution of selectively excited states near the C 1s thresholds in the ESCA molecule by interpreting measured AM spectra via calculations of the core, intermediate, and valence molecular orbitals and transition rates for all final states. The core-excited states show a high degree of selectivity, and the ESCA molecule showcases this in both the site- and state-selective population of AM states. The calculation, utilizing EOM-CCSD to obtain Dyson orbitals and Coulomb waves to describe outgoing photoelectrons, shows very good agreement with the experimental valence electron spectrum, supporting the continued use of this method for photoelectron spectroscopy. We find that several transitions exhibit preferential enhancement upon excitation of certain inner-shell electrons and motivate the investigation of two features for excitation at the COO and CF_3_ sites by RASSCF/PT2 and approximate population analysis of AM intensities. We show that a low-level theory for the intensity, which adds no additional cost to the electronic structure calculation by neglecting the continuum wave function, sufficiently captures enhancement features in resonant AM decay. Therefore, this approach is well suited to simulating AM decay spectra across multiple structures from dynamics simulations. The results reveal a high sensitivity of the resonant AM decay signal when both the core-excited and the final state hole orbitals are spatially local to the absorbing atom site. This enables the possibility for resonant AM decay to be a functional group selective probe in future time-resolved dynamics studies of polyatomic molecules. Recently, time-resolved AM spectroscopy was proposed to settle a debate on the mechanism of ultrafast internal conversion in the ethylene cation to provide a more structurally sensitive probe than previous XAS studies.^13,42^ Future theoretical studies may verify our findings by employing the one-center approximation, which evaluates absolute decay rates with atomic integrals and can produce results in good agreement with experiment.^33^ Furthermore, the inclusion of dynamic correlation via PT2 is a major computational bottleneck and prevents a full-spectrum decay calculation. Multiconfigurational pair-density functional theory^43^ adds dynamic correlation to RASSCF wave functions with minimal additional computational overhead. Future studies using these methods will enable a more comprehensive analysis of larger systems and excited-state dynamics experiments.^12^

## Supporting Information Available

Additional information on the calculation of the valence photoelectron spectrum and the resonant AM spectra; details on the scaling of the different contributions to the resonant spectra; calculated results on the valence photoelectron spectrum with EOM-CCSD Dyson orbital norms, the resonant AM spectrum with spectator decay channels, and molecular orbitals for the final 1-hole states (PDF)

## References

[ref1] Travnikova O.; Bo̷rve K. J.; Patanen M.; Söderström J.; Miron C.; Sæthre L. J.; Mårtensson N.; Svensson S. The ESCA molecule—Historical remarks and new results. J. Electron Spectrosc. Relat. Phenom. 2012, 185, 191–197. 10.1016/j.elspec.2012.05.009.

[ref2] Swallow J. E. N.; Jones E. S.; Head A. R.; Gibson J. S.; David R. B.; Fraser M. W.; van Spronsen M. A.; Xu S.; Held G.; Eren B.; Weatherup R. S. Revealing the Role of CO during CO_2_ Hydrogenation on Cu Surfaces with In Situ Soft X-Ray Spectroscopy. J. Am. Chem. Soc. 2023, 145, 6730–6740. 10.1021/jacs.2c12728. 36916242 PMC10064333

[ref3] Andersson T.; Zhang C.; Björneholm O.; Mikkelä M.-H.; Jänkälä K.; Anin D.; Urpelainen S.; Huttula M.; Tchaplyguine M. Electronic structure transformation in small bare Au clusters as seen by x-ray photoelectron spectroscopy. Journal of Physics B: Atomic, Molecular and Optical Physics 2017, 50, 015102 10.1088/1361-6455/50/1/015102.

[ref4] Iwayama H.; Sisourat N.; Lablanquie P.; Penent F.; Palaudoux J.; Andric L.; Eland J. H. D.; Bučar K.; Žitnik M.; Velkov Y.; Hikosaka Y.; Nakano M.; Shigemasa E. A local chemical environment effect in site-specific Auger spectra of ethyl trifluoroacetate. J. Chem. Phys. 2013, 138, 024306 10.1063/1.4773294. 23320682

[ref5] Inhester L.; Oostenrijk B.; Patanen M.; Kokkonen E.; Southworth S. H.; Bostedt C.; Travnikova O.; Marchenko T.; Son S.-K.; Santra R.; Simon M.; Young L.; Sorensen S. L. Chemical Understanding of the Limited Site-Specificity in Molecular Inner-Shell Photofragmentation. J. Chem. Phys. Lett. 2018, 9, 1156–1163. 10.1021/acs.jpclett.7b03235. 29444399

[ref6] Sorensen S. L.; Zheng X.; Southworth S. H.; Patanen M.; Kokkonen E.; Oostenrijk B.; Travnikova O.; Marchenko T.; Simon M.; Bostedt C.; Doumy G.; Cheng L.; Young L. From synchrotrons for XFELs: the soft x-ray near-edge spectrum of the ESCA molecule. Journal of Physics B: Atomic, Molecular and Optical Physics 2020, 53, 244011 10.1088/1361-6455/abc6bd.

[ref7] Rennie E. E.; Hergenhahn U.; Kugeler O.; Rüdel A.; Marburger S.; Bradshaw A. M. A core-level photoionization study of furan. J. Chem. Phys. 2002, 117, 6524–6532. 10.1063/1.1504435.

[ref8] Piancastelli M. N.; Céolin D.; Travnikova O.; Bao Z.; Hoshino M.; Tanaka T.; Kato H.; Tanaka H.; Harries J. R.; Tamenori Y.; Prümper G.; Lischke T.; Liu X. J.; Ueda K. A high-resolution study of resonant Auger decay processes in N2O after core electron excitation from terminal nitrogen, central nitrogen and oxygen atoms to the 3π LUMO. Journal of Physics B: Atomic, Molecular and Optical Physics 2007, 40, 3357 10.1088/0953-4075/40/17/004.

[ref9] Bolognesi P.; O’Keeffe P.; Ovcharenko Y.; Avaldi L.; Carravetta V. Resonant Auger spectroscopy at the carbon and nitrogen K-edges of pyrimidine. J. Chem. Phys. 2012, 136, 154308 10.1063/1.4704893. 22519327

[ref10] Holzmeier F.; Wolf T. J. A.; Gienger C.; Wagner I.; Bozek J.; Nandi S.; Nicolas C.; Fischer I.; Gühr M.; Fink R. F. Normal and resonant Auger spectroscopy of isocyanic acid, HNCO. J. Chem. Phys. 2018, 149, 034308 10.1063/1.5030621. 30037265

[ref11] de Moura C. E. V.; Laurent J.; Bozek J.; Briant M.; Çarçabal P.; Cubaynes D.; Shafizadeh N.; Simon M.; Soep B.; Püttner R.; Goldsztejn G. Experimental and theoretical study of resonant core-hole spectroscopies of gas-phase free-base phthalocyanine. Phys. Chem. Chem. Phys. 2023, 25, 15555–15566. 10.1039/D3CP01746J. 37252735

[ref12] Wolf T. J. A.; et al. Transient resonant Auger–Meitner spectra of photoexcited thymine. Faraday Discuss. 2021, 228, 555–570. 10.1039/D0FD00112K. 33566045

[ref13] Cabral Tenorio B. N.; Pedersen J.; Barbatti M.; Decleva P.; Coriani S. Auger–Meitner and X-ray Absorption Spectra of Ethylene Cation: Insight into Conical Intersection Dynamics. J. Phys. Chem. A 2024, 128, 107–117. 10.1021/acs.jpca.3c06386. 38134450

[ref14] Wang C.; Gong M.; Zhao X.; Nan Q. W.; Yu X. Y.; Cheng Y.; Kimberg V.; Liu X.-J.; Vendrell O.; Ueda K.; Zhang S. B. Rebuilding the vibrational wavepacket in TRAS using attosecond X-ray pulses. Commun. Phys. 2024, 7, 1 10.1038/s42005-023-01507-3.

[ref15] Preobrajenski A.; Generalov A.; Öhrwall G.; Tchaplyguine M.; Tarawneh H.; Appelfeller S.; Frampton E.; Walsh N. FlexPES: A versatile soft x-ray beamline at the MAX IV Laboratory. J. Synchrotron Radiat. 2023, 30, 831 10.1107/S1600577523003429. 37159290 PMC10325024

[ref16] Bässler M.; Forsell J. O.; Björneholm O.; Feifel R.; Jurvansuu M.; Aksela S.; Sundin S.; Sorensen S. L.; Nyholm R.; Ausmees A.; Svensson S. Soft X-ray undulator beam line I411 at MAX-II for gases, liquids and solid samples. J. Electron Spectrosc. Relat. Phenom. 1999, 101–103, 953 10.1016/S0368-2048(98)00379-X.

[ref17] Pulkkinen H.; Aksela S.; Sairanen O.-P.; Hiltunen A.; Aksela H. Correlation effects in the - MM Auger transitions of Ar. Journal of Physics B: Atomic, Molecular and Optical Physics 1996, 29, 3033–3050. 10.1088/0953-4075/29/14/016.

[ref18] Stanton J. F.; Bartlett R. J. The equation of motion coupled-cluster method. A systematic biorthogonal approach to molecular excitation energies, transition probabilities, and excited state properties. J. Chem. Phys. 1993, 98, 7029–7039. 10.1063/1.464746.

[ref19] Matthews D. A.; Cheng L.; Harding M. E.; Lipparini F.; Stopkowicz S.; Jagau T.-C.; Szalay P. G.; Gauss J.; Stanton J. F. Coupled-cluster techniques for computational chemistry: The CFOUR program package. J. Chem. Phys. 2020, 152, 214108 10.1063/5.0004837. 32505146

[ref20] Melania Oana C.; Krylov A. I. Dyson orbitals for ionization from the ground and electronically excited states within equation-of-motion coupled-cluster formalism: Theory, implementation, and examples. J. Chem. Phys. 2007, 127, 234106 10.1063/1.2805393. 18154374

[ref21] Gozem S.; Krylov A. I. The ezSpectra suite: An easy-to-use toolkit for spectroscopy modeling. Wiley Interdisciplinary Reviews: Computational Molecular Science 2022, 12, e1546 10.1002/wcms.1546.

[ref22] Oana C. M.; Krylov A. I. Cross sections and photoelectron angular distributions in photodetachment from negative ions using equation-of-motion coupled-cluster Dyson orbitals. J. Chem. Phys. 2009, 131, 124114 10.1063/1.3231143. 19791859

[ref23] Gozem S.; Gunina A. O.; Ichino T.; Osborn D. L.; Stanton J. F.; Krylov A. I. Photoelectron Wave Function in Photoionization: Plane Wave or Coulomb Wave?. J. Phys. Chem. Lett. 2015, 6, 4532–4540. 10.1021/acs.jpclett.5b01891. 26509428

[ref24] Hudock H. R.; Martínez T. J. Excited-State Dynamics of Cytosine Reveal Multiple Intrinsic Subpicosecond Pathways. ChemPhysChem 2008, 9, 2486–2490. 10.1002/cphc.200800649. 19006165

[ref25] Ruckenbauer M.; Mai S.; Marquetand P.; González L. Revealing Deactivation Pathways Hidden in Time-Resolved Photoelectron Spectra. Sci. Rep. 2016, 6, 35522 10.1038/srep35522. 27762396 PMC5071879

[ref26] Moitra T.; Coriani S.; Decleva P. Capturing Correlation Effects on Photoionization Dynamics. J. Chem. Theory Comput. 2021, 17, 5064–5079. 10.1021/acs.jctc.1c00303. 34254803

[ref27] Fdez. Galván I.; et al. OpenMolcas: From Source Code to Insight. J. Chem. Theory Comput. 2019, 15, 5925–5964. 10.1021/acs.jctc.9b00532. 31509407

[ref28] Werner H.-J.; Meyer W. A quadratically convergent MCSCF method for the simultaneous optimization of several states. J. Chem. Phys. 1981, 74, 5794–5801. 10.1063/1.440892.

[ref29] Malmqvist P. Å.; Rendell A.; Roos B. O. The restricted active space self-consistent-field method, implemented with a split graph unitary group approach. J. Phys. Chem. 1990, 94, 5477–5482. 10.1021/j100377a011.

[ref30] Malmqvist P. Å.; Pierloot K.; Shahi A. R. M.; Cramer C. J.; Gagliardi L. The restricted active space followed by second-order perturbation theory method: Theory and application to the study of CuO2 and Cu2O2 systems. J. Chem. Phys. 2008, 128, 204109 10.1063/1.2920188. 18513012

[ref31] Grell G.; Kühn O.; Bokarev S. I. Multireference quantum chemistry protocol for simulating autoionization spectra: Test of ionization continuum models for the neon atom. Phys. Rev. A 2019, 100, 042512 10.1103/PhysRevA.100.042512.

[ref32] Grell G.; Bokarev S. I. Multi-reference protocol for (auto) ionization spectra: Application to molecules. J. Chem. Phys. 2020, 152, 074108 10.1063/1.5142251. 32087635

[ref33] Tenorio B. N. C.; Voß T. A.; Bokarev S. I.; Decleva P.; Coriani S. Multireference Approach to Normal and Resonant Auger Spectra Based on the One-Center Approximation. J. Chem. Theory Comput. 2022, 18, 4387–4407. 10.1021/acs.jctc.2c00252. 35737643 PMC9281372

[ref34] Mitani M.; Takahashi O.; Saito K.; Iwata S. Theoretical molecular Auger spectra with electron population analysis. Journal of electron spectroscopy and related phenomena 2003, 128, 103–117. 10.1016/S0368-2048(02)00270-0.

[ref35] Tashiro M.; Ueda K.; Ehara M. Auger decay of molecular double core-hole state. J. Chem. Phys. 2011, 135, 154307 10.1063/1.3651082. 22029313

[ref36] Fouda A. E. A.; Koulentianos D.; Young L.; Doumy G.; Ho P. J. Resonant double-core excitations with ultrafast, intense X-ray pulses. Mol. Phys. 2022, e2133749.

[ref37] Deleuze M. S.; Trofimov A. B.; Cederbaum L. S. Valence one-electron and shake-up ionization bands of polycyclic aromatic hydrocarbons. I. Benzene, naphthalene, anthracene, naphthacene, and pentacene. J. Chem. Phys. 2001, 115, 5859–5882. 10.1063/1.1386414.

[ref38] Carnovale F.; Gan T.; Peel J. Semi-empirical calculations and the assignment of valence photoelectron spectra of large molecules: Phenalen-9-amino-1-imine. J. Electron Spectrosc. Relat. Phenom. 1979, 15, 173–176. 10.1016/0368-2048(79)87029-2.

[ref39] Trofimov A. B.; Schirmer J.; Kobychev V. B.; Potts A. W.; Holland D. M. P.; Karlsson L. Photoelectron spectra of the nucleobases cytosine, thymine and adenine. Journal of Physics B: Atomic, Molecular and Optical Physics 2006, 39, 305 10.1088/0953-4075/39/2/007.

[ref40] Zaytseva I. L.; Trofimov A. B.; Schirmer J.; Plekan O.; Feyer V.; Richter R.; Coreno M.; Prince K. C. Theoretical and Experimental Study of Valence-Shell Ionization Spectra of Guanine. J. Phys. Chem. A 2009, 113, 15142–15149. 10.1021/jp905299z. 20028182

[ref41] Geng T.; Ehrmaier J.; Schalk O.; Richings G. W.; Hansson T.; Worth G.; Thomas R. D. Time-Resolved Photoelectron Spectroscopy Studies of Isoxazole and Oxazole. J. Phys. Chem. A 2020, 124, 3984–3992. 10.1021/acs.jpca.9b11788. 32242664 PMC7304896

[ref42] Zinchenko K. S.; Ardana-Lamas F.; Seidu I.; Neville S. P.; van der Veen J.; Lanfaloni V. U.; Schuurman M. S.; Wörner H. J. Sub-7-fs conical-intersection dynamics probed at the carbon K-edge. Science 2021, 371, 489–494. 10.1126/science.abf1656. 33510022

[ref43] Li Manni G.; Carlson R. K.; Luo S.; Ma D.; Olsen J.; Truhlar D. G.; Gagliardi L. Multiconfiguration Pair-Density Functional Theory. J. Chem. Theory Comput. 2014, 10, 3669–3680. 10.1021/ct500483t. 26588512

