# Supplementary material for: Influence of Selective Carbon 1s Excitation on Auger–Meitner Decay in the ESCA Molecule
Source: J Phys Chem Lett. 2024 Apr 12;15(16):4286–93. doi: 10.1021/acs.jpclett.3c03611 (PMC11057383; doi:10.1021/acs.jpclett.3c03611)
Supplement: Supplementary file 1 — jz3c03611_si_001.pdf [file jz3c03611_si_001.pdf]

Supplementary Material: The Influence of Selective Carbon 1s Excitation on Auger-Meitner Decay in the ESCA Molecule

A.E.A.Fouda,[†,‡] V. Lindblom,[¶] S.H. Southworth,[†] G. Doumy,[†] P. J. Ho,[†] L. Young,[†,‡] L. Cheng,[§] and S. L. Sorensen[*,¶]

†*Chemical Sciences and Engineering Division, Argonne National Laboratory, 9700 S. Cass Avenue, Lemont, IL 60439, USA*

‡*Department of Physics and James Franck Institute, The University of Chicago, Chicago, IL 60637, USA*

¶*Department of Physics, Lund University, Box 118, 22100 Lund, Sweden*

§*Department of Chemistry, Johns Hopkins University, Baltimore, MD 21218 USA*

E-mail: stacey.sorensen@sljus.lu.se,adamfouda@uchicago.edu

Calculation Details

All calculations were performed using a ground state geometry optimized in our previous studies[1,2] and no point-group symmetry restrictions were applied. All 1D theoretical spectra shown are fitted with a Gaussian broadening of 1.0 eV FWHM. The 2D theoretical map was fitted with 1.25 eV FWHM 2D Gaussians in the x and y axis.

Valence Photoelectron Spectra

EOM-CCSD Dyson orbitals from the CFOUR program[3] were used to describe the bound states of the photoionization calculation. The continuum electron wavefunction and cross section calculations were performed using the ezDyson software.[4,5] The latter analytically solves the radial Schrödinger equation for the continuum electron by using a point charge to describe the interaction between the outgoing electron and remaining core. A point charge of +1 is centered at the centroid of the Dyson orbital in the radial Coulomb wave description of the continuum electron. The photoionization cross section was averaged over the molecular orientation with linearly polarised pulses on a Cartesian grid with 301 points between -15 and +15 Å in the x, y and z planes. The maximum angular momentum quantum number used in the Coulomb waves was $l_{max} = 10$.

Resonant Auger-Meitner Spectra

The OpenMolcas electronic structure code[6] with local modification for printing MO Mulliken populations was used for the RASSCF/PT2 calculations in this work. Separate calculations of the decay to the participator ($1h$) and spectator ($2h1p$) final bound states were carried out, using different active spaces to capture the necessary final states at a practical computational cost. Here we use the RASSCF(n,l,m;i,j,k) notation to describe the active spaces, where n, l and m refer to the total number of active electrons, the maximum number of holes allowed in RAS1 and the maximum number of electrons allowed in RAS3 respectively; i, j, and k refer to the number of orbitals in the RAS1, RAS2 and RAS3 subspaces.

In the $1h$ calculation, the ground and core-excited states were calculated at the single state- (SS-) RASPT2(56,1,1;1,27,1) level of theory, the valence cation states were calculated at the SS-RASPT2(55,1,0;1,27,1) level of theory and the RASSCF calculation was averaged over 27 states. Restricting the number of electrons in RAS3 to zero ensures the calculation includes all possible valence $1h$ states. Either the CF_3 or COO carbon 1s was placed into RAS1 with the highly excited state (HEXS) scheme[7] applied and placed into its own individ-

ual sub-symmetry group to prevent orbital rotation during the optimization to generate the core-excited state. The remaining 1s orbitals were kept frozen and all 27 occupied valence orbitals were placed into RAS2. In each case, the virtual orbital involved in the core-excitation was placed into RAS3. For both the COO $1s \rightarrow \pi^*$ and CF_3 $1s \rightarrow \pi$ excitations this was the LUMO. For the CF_3 $1s \rightarrow \sigma^*$ excitation this was the LUMO+1, which was swapped with the LUMO in order for it to be placed into RAS3. The single state RASPT2 calculation used an imaginary shift of 0.1 a.u.

In the $2h1p$ calculation, the ground, core-excited and valence cation states were calculated at the single SS-RASPT2(38,1,1;1,18,1) level of theory. The final cation state calculation was state averaged over 379 states. In order to reduce the computational expense, the 9 highest binding energy orbitals after the 1s orbital were not included in the active space and optimized at the Hartree-Fock level of theory. The rest of the calculation was identical to the $1h$, except that now both the orbitals in RAS1 and RAS3 were placed into their own individual sub-symmetry groups to prevent orbital rotation during the optimization. Furthermore, the single state RASPT2 calculation used an imaginary shift of 0.4 a.u. to prevent inclusion of intruder states.

To adopt an efficient approach for the basis set in Auger-Meitner decay calculations all calculations use the cc-pVTZ basis set on the core-hole containing atom; all other atoms used the cc-pVDZ basis set.[8] This was applied to both the ground and valence cation calculations for a particular spectrum as well. Furthermore, all ground, core-excited and valence cation calculations for the spectra were calculated from the Hartree-Fock guess orbitals for the given basis set. No global point group symmetry restrictions were applied.

The Auger-Meitner intensity is approximated by the electronic populations of the molecular orbitals on the core-hole atom in the core-excited state wavefunction and is described in detail elsewhere.[9–11] In this work our calculations use the diagonal approximation used by Mitani and coworkers.[9]

Scaling Factor for Theoretical Valence Photoelectron and Resonant Auger-Meitner Spectra

In order to correctly account for the competing core-excitation and direct valence ionization processes in the calculation, both theoretical contributions were separately normalized to 1. Then the resonant Auger-Meitner spectra were scaled by a factor (β), derived from the ratio of the total valence ezDyson photoelectron cross section and the EOM-CCSD core-excitation cross section from previous calculations.[1] The formula and values involved in computing the scaling factor are given by

$$\beta = \frac{\sigma_{Tot.}}{\sigma^{XAS}}, \tag{1}$$

where,

$$\sigma_{Tot.} = \sum_i \sigma_i^{PES}. \tag{2}$$

σ^{XAS} is derived from the core-excitation oscillator strength (F) using the following formula,[12] which considers a resonant pulse with a bandwidth (≈ 0.05 eV) significantly narrower than the lifetime broadening of the core-excited states ($\gamma \approx 0.1$ eV, based on the molecular carbon K-shell ionization linewidth[13]),

$$\sigma^{XAS} = \frac{4\pi\alpha F}{\gamma}. \tag{3}$$

Table. S1 provides the values for F, $\sigma_{Tot.}$ and β used in this work.

Table S1: Table of experimental energies, core excitation oscillator strengths (F),[1] total valence direct photoionization cross section ($\sigma_{Tot.}$) and scaling factors (β).

Resonance	E_{exp} (eV)	F (a.u.)	Val. $\sigma_{Tot.}$ ($a.u.$)	β
COO $1s \rightarrow \pi^*$	288.30	0.066	0.70	0.42
CF_3 $1s \rightarrow \pi$	293.33	0.039	0.67	0.69
CF_3 $1s \rightarrow \sigma^*$	296.50	0.084	0.66	0.32

Results

Dyson Norm Valence Photoelectron Spectra

Fig. S1 shows the good experimental agreement of the sudden approximation (SA) spectra, which neglect the outgoing electron entirely by approximating the relative cross section with the Dyson orbital norm values presented in the main text.

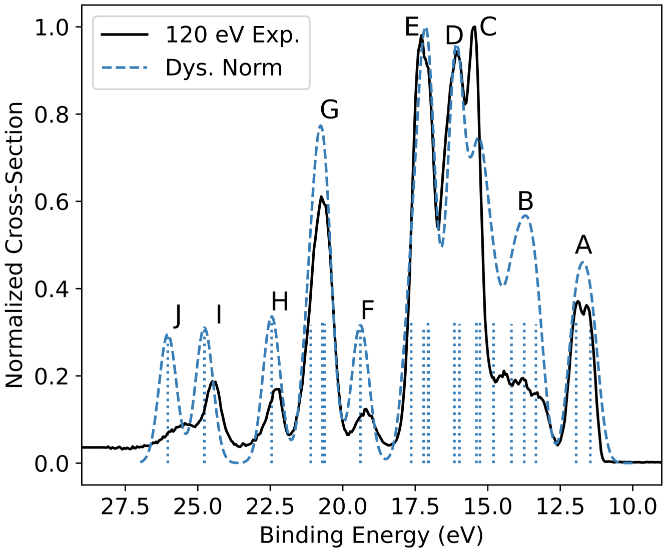

Figure S1: Comparison between experiment and theory for the direct valence photoionization spectrum of the ESCA molecule. The EOM-CSSD calculated spectra with Dyson orbital norms (SA) is compared to the 120 eV experimental spectrum. Peaks have been labelled **A**-**J** and the calculated ionization energies, Dyson orbital norms and cross sections are given in the main text.

Resonant Spectator Enhancement

In Fig. S2 we include the $2h1p$ final states to the theoretical resonant spectra shown in the main text. In-order to achieve agreement in Fig. S2 calculated spectator intensities were scaled by a factor of 3.5. This is due to an over-estimation of the these intensities with respect to the calculated participator intensities. This is likely to arise from the Auger-Meitner intensity calculation neglecting the continuum wavefunction of the ejected electron and that different active spaces were used for the participator and spectator calculations.

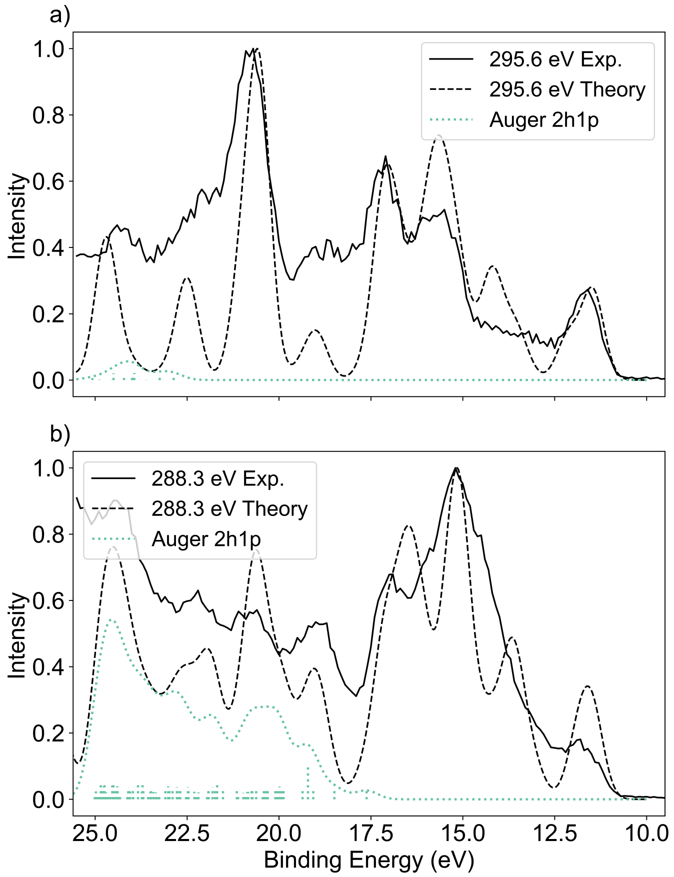

Figure S2: Resonant experimental spectra (black solid) and theoretical spectra (dashed black) which are a combination of the RASSCF/PT2 calculated Auger-Meitner participator ($1h$) and spectator ($2h1p$) channels (green dotted) and the EOM-CCSD ezDyson calculated direct photoionization (PES $1h$) channels. A scaling factor of 3.5 was applied to the calculated spectator intensities at 288.3 eV (COO). The calculated Auger $1h$ spectra were shifted by the values reported in the main text and the $2h1p$ spectra were shifted by 0.80 eV at 288.3 eV and 0.82 eV for 295.6 eV.

Molecular Orbital Tables

Molecular orbital analysis of the $1h$ final states are collected in Figs. S3-S5. The left column shows the Dyson orbitals from the EOM-CCSD calculation used to generate the direct PES spectra in the ezDyson Software. The middle and right columns show the natural hole orbitals from the RASSCF calculations used in the COO $1s \rightarrow \pi^*$ and COO $1s \rightarrow \pi^*$ Auger calculations respectively. In all cases the calculated binding energies are given. For the Auger-Meitner $1h$ final states, there are subtle differences in the shape, order and binding energy despite the calculations being done at the same level of theory SA-RASSCF/SS-RASPT2(55,1,0;1,27,1) due to the different $1s$ orbital in the RAS1 active subspace of each calculation. The reported RASPT2 binding energies have been shifted by 0.05 eV and 0.8 eV for COO $1s \rightarrow \pi^*$ and CF_3 $1s \rightarrow \sigma^*$ respectively, to correspond with the calculated spectra.

References

(1) Sorensen, S. L.; Zheng, X.; Southworth, S. H.; Patanen, M.; Kokkonen, E.; Oostenrijk, B.; Travnikova, O.; Marchenko, T.; Simon, M.; Bostedt, C.; Doumy, G.; Cheng, L.; Young, L. From synchrotrons for XFELs: the soft x-ray near-edge spectrum of the ESCA molecule. *Journal of Physics B: Atomic, Molecular and Optical Physics* **2020**, *53*, 244011.

(2) Zheng, X.; Cheng, L. Performance of Delta-Coupled-Cluster Methods for Calculations of Core-Ionization Energies of First-Row Elements. *Journal of Chemical Theory and Computation* **2019**, *15*, 4945–4955, PMID: 31365823.

(3) Matthews, D. A.; Cheng, L.; Harding, M. E.; Lipparini, F.; Stopkowicz, S.; Jagau, T.-C.; Szalay, P. G.; Gauss, J.; Stanton, J. F. Coupled-cluster techniques for computational chemistry: The CFOUR program package. *The Journal of Chemical Physics* **2020**, *152*, 214108.

(4) Gozem, S.; Gunina, A. O.; Ichino, T.; Osborn, D. L.; Stanton, J. F.; Krylov, A. I. Photoelectron Wave Function in Photoionization: Plane Wave or Coulomb Wave? *The Journal of Physical Chemistry Letters* **2015**, *6*, 4532–4540, PMID: 26509428.

(5) Gozem, S.; Krylov, A. I. The ezSpectra suite: An easy-to-use toolkit for spectroscopy modeling. *Wiley Interdisciplinary Reviews: Computational Molecular Science* **2022**, *12*, e1546.

(6) Fdez. Galván, I. et al. OpenMolcas: From Source Code to Insight. *Journal of Chemical Theory and Computation* **2019**, *15*, 5925–5964, PMID: 31509407.

(7) Delcey, M. G.; Sørensen, L. K.; Vacher, M.; Couto, R. C.; Lundberg, M. Efficient calculations of a large number of highly excited states for multiconfigurational wavefunctions. *Journal of computational chemistry* **2019**, *40*, 1789–1799.

(8) Tenorio, B. N. C.; Voß, T. A.; Bokarev, S. I.; Decleva, P.; Coriani, S. Multireference Approach to Normal and Resonant Auger Spectra Based on the One-Center Approximation. *Journal of Chemical Theory and Computation* **2022**, *18*, 4387–4407, PMID: 35737643.

(9) Mitani, M.; Takahashi, O.; Saito, K.; Iwata, S. Theoretical molecular Auger spectra with electron population analysis. *Journal of electron spectroscopy and related phenomena* **2003**, *128*, 103–117.

(10) Tashiro, M.; Ueda, K.; Ehara, M. Auger decay of molecular double core-hole state. *The Journal of chemical physics* **2011**, *135*, 154307.

(11) Fouda, A. E. A.; Koulentianos, D.; Young, L.; Doumy, G.; Ho, P. J. Resonant double-core excitations with ultrafast, intense X-ray pulses. *Molecular Physics* **2022**, *0*, e2133749.

(12) Ho, P. J. et al. The role of transient resonances for ultra-fast imaging of single sucrose nanoclusters. *Nature Communications* **2020**, *11*, 167.

(13) Carroll, T. X.; Børve, K. J.; Sæthre, L. J.; Bozek, J. D.; Kukk, E.; Hahne, J. A.; Thomas, T. D. Carbon 1s photoelectron spectroscopy of CF4 and CO: Search for chemical effects on the carbon 1s hole-state lifetime. *The Journal of Chemical Physics* **2002**, *116*, 10221–10228.

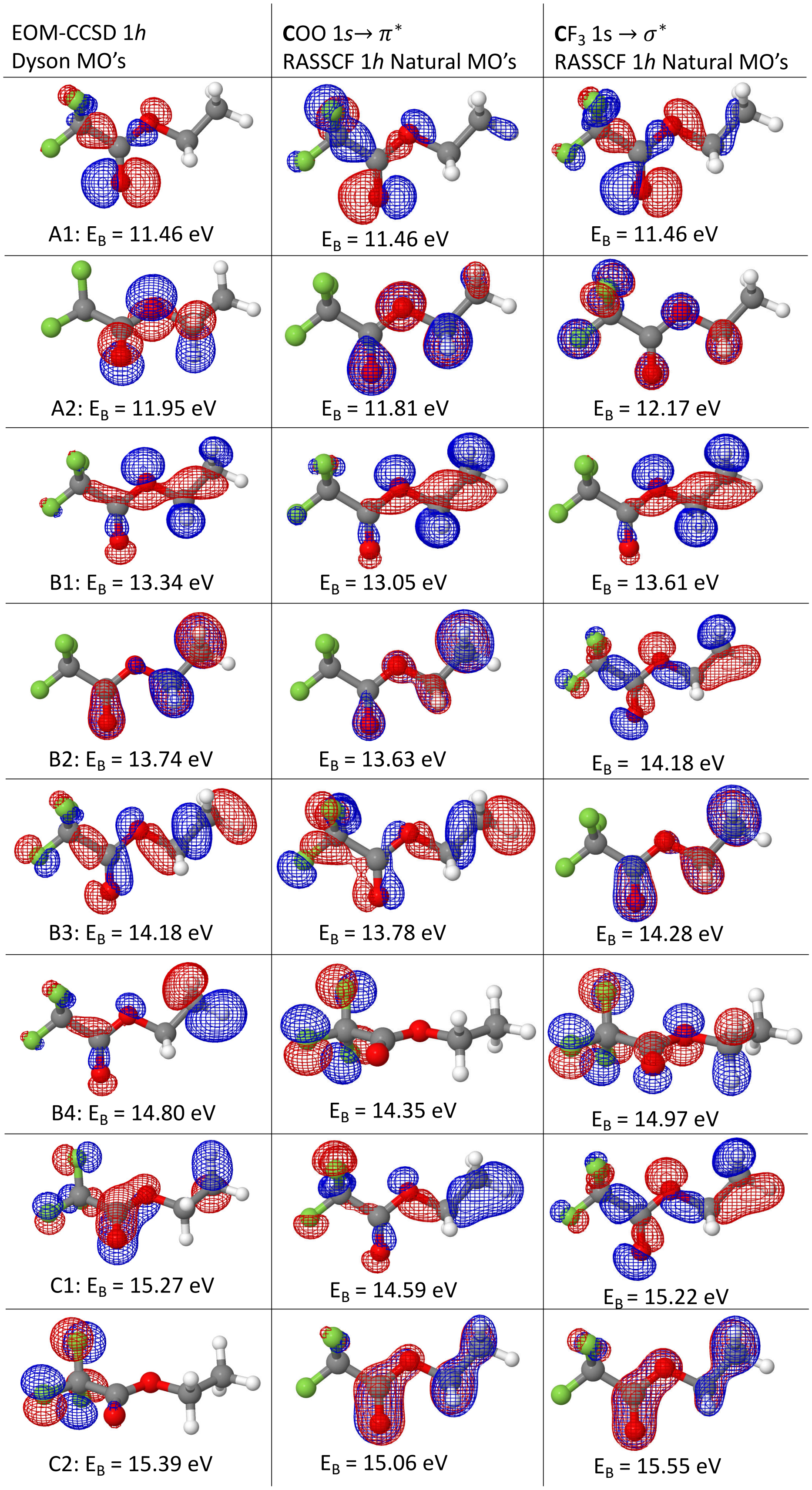

Figure S3: Molecular orbital analysis of the $1h$ final states.

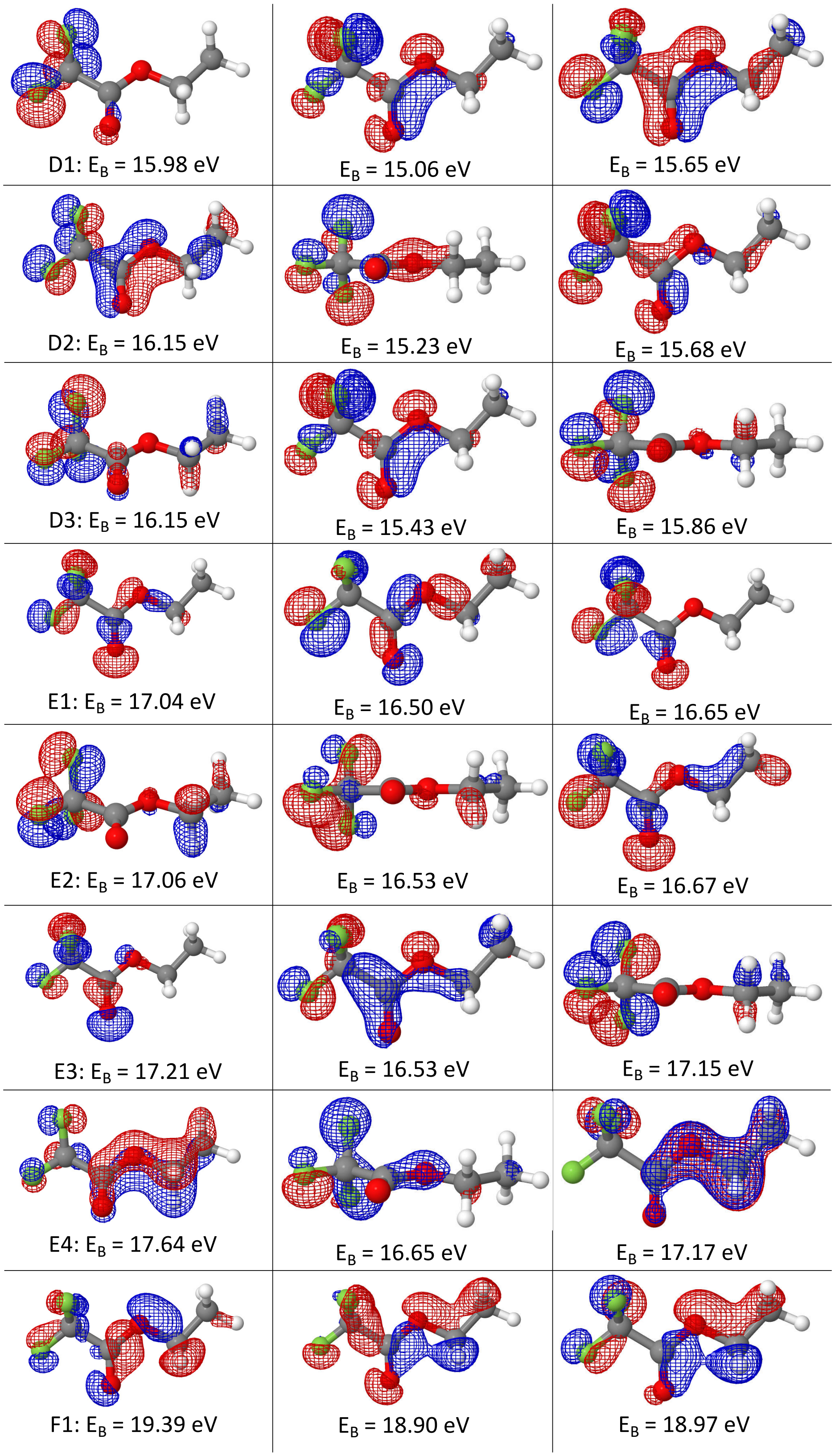

Figure S4: Molecular orbital analysis of the $1h$ final states.

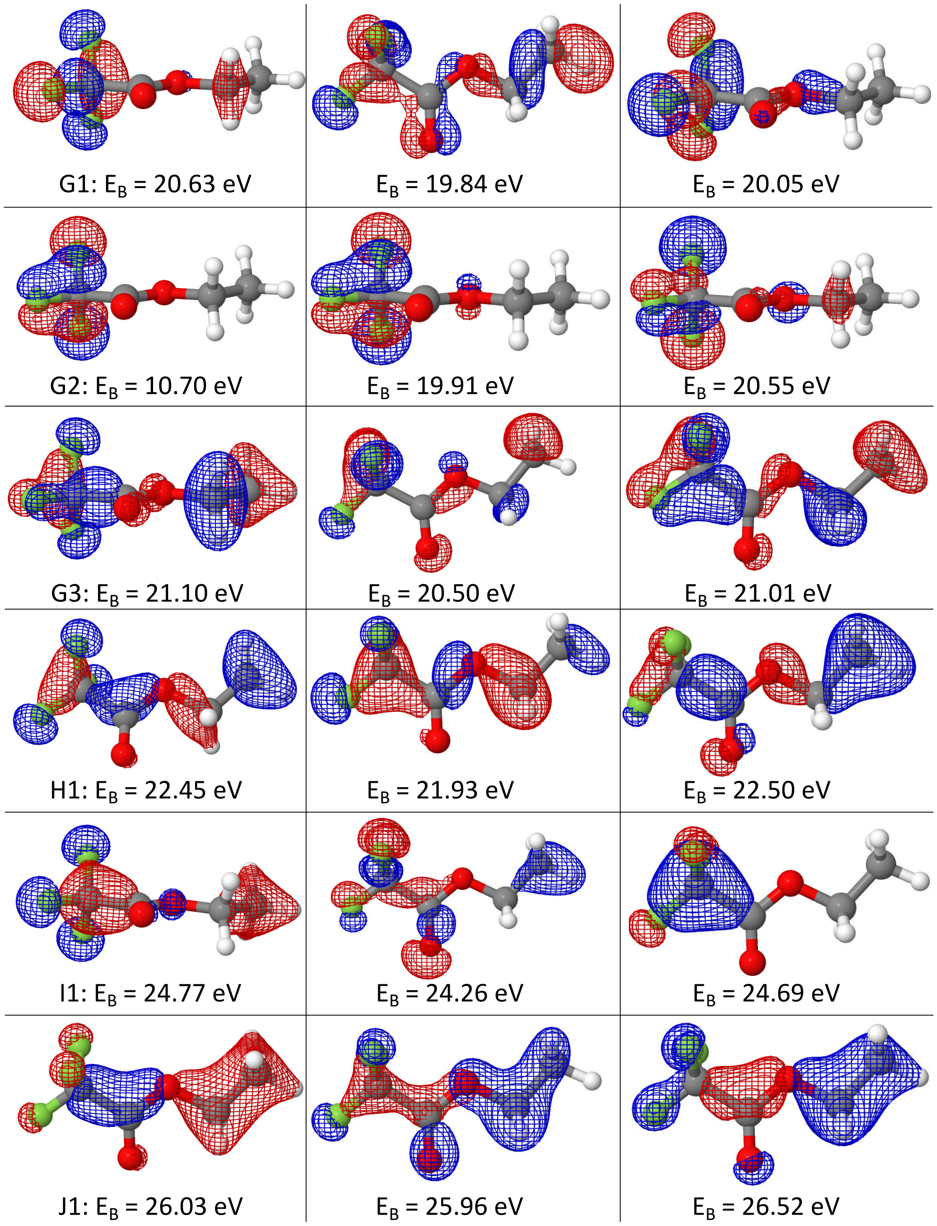

Figure S5: Molecular orbital analysis of the $1h$ final states.
